# *β*-Human Chorionic Gonadotropin Dynamics in Early Gestational Events: A Practical and Updated Reappraisal

**DOI:** 10.1155/2024/8351132

**Published:** 2024-03-07

**Authors:** Demetrio Larraín, Javier Caradeux

**Affiliations:** Department of Obstetrics and Gynecology, Clínica Santa María, Santiago, Chile

## Abstract

In the last decade, the widespread use of transvaginal ultrasound and the availability of highly specific serum assays of human chorionic gonadotropin (hCG) have become mainstays in the evaluation of early pregnancy. These tests have revolutionized the management of pregnancies of unknown location and markedly reduced the morbidity and mortality associated with the misdiagnosis of ectopic pregnancy. However, despite several advances, their misuse and misinterpretations are still common, leading to an increased use of healthcare resources, patient misinformation, and anxiety. This narrative review aims to succinctly summarize the *β*-hCG dynamics in early gestation and provide general gynecologists a practical approach to patients with first-trimester symptomatic pregnancy.

## 1. Introduction

In the last decade, the diagnosis and management of suspected early pregnancy failure (pain, bleeding) have changed dramatically. The widespread availability of transvaginal ultrasound (TVUS) and highly specific assays to measure the serum concentration of human chorionic gonadotropin (hCG) have become mainstays in the evaluation of early pregnancy problems [1]. These tests, which allow early detection of pregnancy and more accurate diagnosis of its complications, have revolutionized the management of intrauterine pregnancy (IUP) and markedly reduced the morbidity and mortality associated with ectopic pregnancy (EP). However, despite the indisputable benefits of these tests, their misuse and misinterpretations are still common, and can lead to severe damage due misdiagnosis of an EP or even to the unintended interruption of a normal IUP [2, 3]. The main objective of this article is to perform a narrative review the *β*-hCG behavior in normal early gestation and to describe the *β*-hCG dynamics in early gestational events. Also, we aimed to summarize the different strategies proposed in cases of pregnancies of unknown location (PUL) and wanted to assess the current value of the so-called “discriminatory zone.” Overall, providing to general gynecologists a practical approach to patients with first-trimester symptomatic gestation.

## 2. hCG: Biochemical and Laboratory Issues

### 2.1. hCG Characteristics

hCG is a glycoprotein, a peptide framework to which carbohydrate side chains are attached. It is a dimeric molecule composed by two subunits called alpha (*α*) and beta (*β*), which are noncovalently linked by disulfide bonds. The *α* subunit is identical to that of other glycoprotein hormones such as follicle stimulating hormone, luteinizing hormone, and thyroid-stimulating hormone. Unique biological activity of each of these hormones is conferred by modifications of the carbohydrate moieties in the *β* subunit [4]. The *β* subunit of hCG is the largest *β* subunit and it is composed by a unique amino acidic tailpiece and more sites for glycosylation than other *β* subunits. These features allow the utilization of highly specific immunoassays for *β*-hCG measurement and confer hCG a longer circulating half-life of approximately 24 hours [4, 5]. Although several human tissues appear to produce hCG, the placenta has the unique ability to glycosylate the protein, thus reducing its metabolism and increasing its biological activity.

During pregnancy, hCG is produced mainly by syncytiotrophoblastic cells [4]. The release of hCG into maternal circulation begins with embryo implantation, 5 to 7 days after fertilization, and is mostly catabolized by the liver, although about 20% is excreted in the urine. To date, its only proven function is to support the corpus luteum [5].

### 2.2. hCG Measurement

Multiple hCG-related molecules are present in serum and urine during pregnancy, including intact active hCG, hyper/hypo-glycosylated hCG, nicked hCG (which is enzymatically cleaved), free *α*/*β* subunits (without biological activity), large free *α*-subunits, nicked free *β*-subunits, and *β*-core fragment (product of the degradation of *β*-subunit in the kidney, and is the principal form of *β*-hCG in urine samples and virtually undetectable in serum). The concentration and mean proportions of such molecules vary widely during pregnancy and among different individuals. Likewise, these isoforms may significantly differ in peptide and carbohydrate structure, and therefore, in their recognition by different *β*-hCG immunoassays [5, 6]. To date, there are many commercial assays available for measuring *β*-hCG concentrations in serum and urine samples that use different antibodies combinations. Such combinations may detect intact hCG molecules, free subunits, nicked hCG molecules, or combinations of them, and are the cause of great interassay heterogeneity [6].

*β*-hCG can be measured quantitatively and qualitatively in serum and urine. While qualitative tests report a positive or negative result, in quantitative tests *β*-hCG levels are reported in mIU/mL or IU/L. Overall, serum testing has a much higher sensitivity and specificity than urine testing, rendering an overall better diagnostic performance [5]. While qualitative urine tests have a sensitivity of 20–25 IU/L, current quantitative serum tests have a sensitivity of less than 10 IU/L. Therefore, quantitative serum *β*-hCG measurement is the method of choice in the evaluation and follow-up of symptomatic early pregnancies [5].

In order to avoid interassay variability, it is highly recommended to use the same assay (or the same laboratory) for all samples. Furthermore, 48 hours sampling intervals are usually recommended during follow-up. Because, after 1 day, the difference between the mean percent *β*-hCG increase of intrauterine and ectopic pregnancies (≅20%) is less than twice both interassay and intra-assay variability (≅15%), and so may be less reliable [7]. Of note, serum *β*-hCG levels have been measured over the years using the First International Reference Preparation (IRP), the Second International Standard (IS), the Third IS, and the Fourth IS. The Second IS yields results that are approximately half the numeric values of the other three methods. In this review, unless otherwise specified, all *β*-hCG values mentioned are referred to the first IRP, third IS, or fourth IS.

### 2.3. *β*-hCG Dynamics in Normal Pregnancy

In spontaneously conceived singleton pregnancy, *β*-hCG becomes detectable in maternal serum as early as 8–10 days after ovulation in normal conception cycles. The maternal circulating *β*-hCG concentration is approximately 50–100 IU/L at the time of expected but missed menses. In general, levels double every 1.4–2.1 days, and a maximal level of about 50,000–100,000 IU/L in the maternal circulation is reached at 8–10 weeks of gestation. Then, *β*-hCG levels decrease to about 10,000–20,000 IU/L by 18–20 weeks of gestation and remain at that level to term.

It is widely accepted that *β*-hCG concentrations rise predictably, at an exponential pace, during early normal IUP. However, the rate of increase slows gradually as maternal age and initial *β*-hCG concentrations augment. For initial *β*-hCG values of less than 1,500 IU/L, 1,500−3,000 IU/L and greater than 3,000 IU/L, the predicted 2-day minimal rise is 49%, 40% and 33%, respectively [8]. For decades, clinicians have relied on a normal “doubling time” to characterize a viable pregnancy when ultrasonography is not available or is nondiagnostic. However, the minimal normal increase in *β*-hCG concentrations for women with a viable IUP has progressively become more conservative (Table 1).

This traditional approach of “doubling time” is based on the conception that *β*-hCG levels should increase at least 66% of the initial value every 48 hours in viable IUPs [7]. However, caution should be taken, as this value was based on the 85% confidence interval of a study conducted in only 20 patients. More recently, Barnhart et al. [9] analyzed the change in serial *β*-hCG levels among 287 women with symptomatic early pregnancies and nondiagnostic ultrasound who ultimately proved to have viable IUPs. Although the median rise in *β*-hCG levels was 50% after 1 day, 124% after 2 days, and 400% after 4 days, the slowest or minimal rise for a normal viable IUP was 24% after 1 day and 53% after 2 days [9].

Interestingly, when the aforementioned *β*-hCG references were applied to a group of 1,249 patients considered at risk of EP, a minimal rise in hCG levels of 35% in 2 days (the lower bound from a 99.9% confidence interval for the rise of *β*-hCG among IUP) was the best to characterize a potentially viable gestation, and further minimize potential interruption of a desired pregnancy [10]. Although 99% of normal gestations will have at least this rise in hCG, this should not be interpreted as a threshold for viability and the diagnosis of a nonviable pregnancy should not be solely based on early *β*-hCG changes [11].  Table 2 summarizes the minimal expected increase in *β*-hCG levels in normal IUPs.

### 2.4. *β*-hCG Dynamics in Pregnancies Conceived through In Vitro Fertilization and Multiple Gestation

The higher risk of EP among patients who conceive using assisted reproductive technologies has already been stated [12]. Therefore, the knowledge and correct interpretation of *β*-hCG patterns in these patients is relevant to avoid delayed diagnosis of EP and to minimize the risk of an unwanted interruption in a highly desired pregnancy. Previous studies have attempted to characterize *β*-hCG dynamics associated with in vitro fertilization (IVF), but most are limited by cross-sectional designs, small sample size and failure to adjust for potentially confounding factors such as multiple embryo transfer and multiple gestations [13, 14]. With respect to *β*-hCG rise among multiple gestations, previous reports have provided conflicting results [14, 15].

In a more recent longitudinal study, Chung et al. [16] characterized the rise of *β*-hCG, and potential factors that could influence the *β*-hCG increase among 391 viable pregnancies achieved by IVF, including 224 singletons, 135 twins and 32 triplets. Multivariate analysis revealed that although absolute *β*-hCG values were significantly higher for twins and triplets, the rates of *β*-hCG rise were comparable to singleton and spontaneous IUPs (average increase of 50% in 1 day and 124% in 2 days). Furthermore, they found that *β*-hCG values were significantly lower in obese patients (BMI > 30), but the rate of increase was similar. Interestingly, the slowest rate of increase in this population of pregnancies known to result in a live birth was 14% in 1 day and 30% in 2 days [16].

The latter confirms that these values are referential, and even so they are useful for clinical management, in most cases they should not be interpreted as a clinical rule, and follow-up is warranted. Likewise, caution should be taken in interpreting *β*-hCG values in IVF-conceived pregnancies complicated by ovarian hyperstimulation syndrome, especially at low *β*-hCG concentrations. Due to extravascular fluid shifts, and hemoconcentration followed post-hydration hemodilution, serum *β*-hCG measurements may be inaccurate.

### 2.5. *β*-hCG Dynamics in Cases of Miscarriage

It is clear that decreasing *β*-hCG values without medical or surgical treatment are indicative of a nonviable pregnancy, either a failed IUP or an EP. The standard rate of *β*-hCG decline that characterizes miscarriage was described 19 years ago. Unlike *β*-hCG increasing patterns, a distinct feature of *β*-hCG declining rates is that the clearance depends on the initial *β*-hCG levels.

In 2004, Barnhart et al. [17] attempted to establish the normal rate of *β*-hCG decline among 710 patients with decreasing *β*-hCG levels who were ultimately diagnosed to have a miscarriage (not requiring medical or surgical intervention). Data were sorted in groups based upon initial *β*-hCG value from 250 IU/L to 5,000 IU/L. The mean days until *β*-hCG level was negligible ranged from 12 to 16. However, they found that higher starting *β*-hCG concentrations were associated with a more rapid decline. The slowest rates of decline for each *β*-hCG group (represented by the 95^th^ centile) ranged from a 21% to 35% reduction in 2 days for initial *β*-hCG values of 250 IU/L and 5,000 IU/L, respectively. After 7 days, the slowest decline rates were 60% and 84% for initial *β*-hCG concentrations of 250 IU/L and 5,000 IU/L, respectively. The former suggests that the “15% drop in 7 days” rule that has been adopted from the studies of medically treated EPs [18] is too conservative to apply for the follow-up of presumed miscarriage. Therefore, for those patients who are being managed expectantly with the hope of spontaneous resolution, a 15% decline in 7 days is slower than expected and should prompt intervention to eliminate the possibility of an EP.

In 2006, another study conducted by the same group, the authors of [19] described the expected rate of *β*-hCG decline in patients with confirmed miscarriage who presented very low initial *β*-hCG levels (<250 IU/L). The authors confirmed that the slowest rates of decline were associated with the lowest initial *β*-hCG values. In their results, the slowest rate of decline for each *β*-hCG group (represented by the 95^th^ centile) ranged from 12% to 21% reduction in 2 days for initial *β*-hCG values of 50 IU/L and 250 IU/L, respectively. Likewise, the slowest rate of decline in 7 days ranged from 34% to 60% for starting *β*-hCG values of 50 IU/L and 250 IU/L, respectively. Therefore, current evidence shows that the natural rate of decline in a failed early pregnancy without intervention is slower than the reported for medical or surgically treated miscarriages [20], probably because of *β*-hCG production by residual trophoblastic tissue [17].

Although *β*-hCG levels that fall along the predicted curves can be managed expectantly, these patients are still at risk of having an EP. Also, levels deemed to decrease too slowly should prompt intervention. Unfortunately, the expected decline cannot be described adequately by a single curve but rather requires a model that differs depending on the initial hCG, and a simple clinical rule is not applicable. Accordingly, a woman with decreasing *β*-hCG values and at risk of EP should be monitored until nonpregnant levels are reached because rupture of an EP can occur while levels are decreasing or are very low [21].  Table 3 summarizes the minimal percentage of decline in *β*-hCG levels for miscarriage according to initial *β*-hCG level.

### 2.6. The Concept of “Pregnancy of Unknown Location”

Pregnancy of unknown location (PUL) is a descriptive term applied to women with a positive pregnancy test who have no evidence of either an IUP, retained products of conception or EP on TVUS [22]. Therefore, this term describes a transient state, and should be considered as a classification, not as a final diagnosis. Furthermore, it is important to note that the concept of PUL is not associated with any *β*-hCG value. Studies from specialized early pregnancy units report PUL rate as low as 8.7% [23], but it is widely accepted that modern units should try to maintain PUL rate < 15% [24].

Although there is consensus that women with a PUL should be followed until a final diagnosis can be made, the PUL-related definitions, diagnostic strategies, possible outcomes, and management vary widely [22]. A usual clinical dilemma is weighing the risk of morbidity due to an EP against the morbidity associated with interventions to achieve a definitive diagnosis and treatment. Therefore, the presence of risk factors for EP should always be assessed in all patients with a PUL.

**Figure 1:**
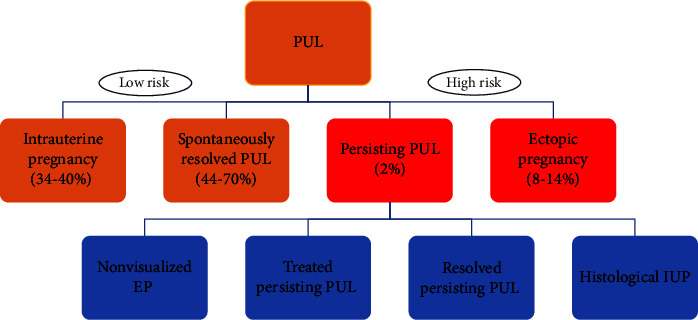
Pregnancies of unknown location (PUL) outcomes based on clinical management.

Albeit the main concern when facing a PUL is the misdiagnosis of EP, only 7–20% of PULs will be diagnosed as EPs [25]. Most cases are subsequently diagnosed with either IUPs that were too early to visualize on the initial TVUS or spontaneously resolving PUL, in which the location of the pregnancy was never confirmed. Whilst the majority will almost certainly be failed IUPs, a proportion will be failed EPs [25]. In 2011, a standardization of PUL nomenclature, definitions, and possible outcomes was proposed [22]. Current categorization of final outcomes of PUL, based on clinical management, is summarized in Figure 1.

### 2.7. PUL Diagnostic Strategies

A patient with a PUL who is clinically stable should at least have repeat TVUS and/or serial measurements of *β*-hCG concentrations, in order to confirm the diagnosis and establish management. A single *β*-hCG measurement cannot diagnose viability or location of a gestation and should not be used for such purpose. Follow-up of a stable patient until achieving a definitive diagnosis of EP is recommended to prevent misdiagnosis and to avoid unnecessary exposure to methotrexate, which can lead to interruption or teratogenicity of an ongoing IUP [3]. Moreover, a definitive location of a PUL cannot always be determined even with an TVUS follow-up because both a miscarriage and an EP may resolve without intervention. Despite many authors have advocated that the use of two serum *β*-hCG concentrations assessed 48 hours apart, expressed as a ratio (*β*-hCG at 48 hours/*β*-hCG at 0 hours), can accurately predict the outcome of women with a PUL [26, 27], most usually follow serial serum *β*-hCG concentrations until these levels deviate from what is expected for a potential viable gestation or a miscarriage.

Two diagnostic strategies in patients with PUL (i.e., at risk for EP) have been proposed. The first, followed in the United Kingdom and Europe, could be considered as more conservative, and relies mainly on ultrasound diagnosis and advocates for a more extended follow-up of PULs without intervention. The second, applied in the United States, could be considered as more aggressive, advocating for interventions, such as uterine evacuation to distinguish a nonviable IUPs from an EP by identifying the presence or absence of chorionic villi [22].

### 2.8. Spontaneously Resolved PUL

Patients with spontaneous resolution of serum *β*-hCG to undetectable levels without medical or surgical intervention are currently classified as spontaneously resolved PUL (formerly failing PUL). This definition considers that the exact location of the gestation is never identified [22]. In clinical practice, the majority of PULs are finally diagnosed as spontaneously resolved PULs [27]. Recently, a multicentric study evaluated the expected rate of *β*-hCG decline in spontaneously resolved PULs among 443 patients using updated statistical methods to generate *β*-hCG elimination curves [28]. In this study, *β*-hCG decline was slower in women who were older than 35 years. Conversely, *β*-hCG decline was faster in women who described pain at presentation compared with those who did not.

As previously reported for spontaneous abortions [9, 10], the rate of *β*-hCG decline was directly proportional to the initial *β*-hCG level. The slowest rates of decline for each hCG group (represented by the 95^th^ centile) ranged from 35% to 50% reduction in 2 days for initial *β*-hCG values of 250 IU/L and 5,000 IU/L, respectively. After 7 days, the slowest decline rates (95^th^ centile) were 66% to 87% for starting *β*-hCG concentrations of 250 IU/L and 5,000 IU/L, respectively [28]. Therefore, the minimal decline in *β*-hCG was faster than previously reported, and a decline slower than these thresholds may indicate the presence of retained trophoblastic tissue or EP. Likewise, an *β*-hCG ratio ≤0.79 or a decrease of *β*-hCG > 21% has been found to be highly accurate for the prediction of a failing PUL [27]. In these cases, no further evaluation is necessary.  Table 4 summarizes the minimal percentage decline in *β*-hCG for spontaneously resolved PUL by initial *β*-hCG level.

### 2.9. PUL Risk Assessment and Management

PUL management has moved away from establishing pregnancy location towards risk assessment of adverse outcomes. Accordingly, outcomes such as EP and persisting PUL are designated as high risk scenarios, while IUP and spontaneously resolved PUL are classified as low risk scenarios [29]. In this way, a more appropriate follow-up arrangement can be made based on the risk of complications.

The majority of asymptomatic hemodynamically stable patients with PUL show a high rate of spontaneous resolution (48–73%) and present a low risk of complications. Therefore, in this group, expectant management has been shown to be safe [30], and most cases are later categorized as a “failed PUL.” Based on these data, some authors have argued that diagnosis of PUL location is an unnecessary cost in low risk patients and suggest that further assessment should be reserved only for women who have been stratified as high risk [31].

On the contrary, others argue for the need of definitive diagnosis by means of uterine evacuation [32], which allows to confirm a miscarriage by the presence of chorionic villi on histopathology. Otherwise, if endometrial biopsy does not contain chorionic villi and/or *β*-hCG levels do not decline after uterine evacuation, the pregnancy is presumed to be extrauterine and can be treated medically with methotrexate [32–34]. The main arguments of such policy are: to restrict the use of methotrexate and its side effects, to allow a shorter time to pregnancy resolution, to bring more accurate fertility counseling and to avoid the need of delaying a subsequent pregnancy due to time required to methotrexate washout [32, 35].

Moreover, it has been shown that empirical treatment of a presumed EP is inaccurate and may result in unnecessary treatment of miscarriages with methotrexate in up to 40% of cases [34]. Likewise, in a cost-effectiveness analysis comparing treatment of presumptive nonviable PULs with methotrexate vs. performing uterine curettage as first step, the use of methotrexate did not decrease costs and was associated with more complications [36].

Although uterine evacuation is widely used in the United States as a diagnostic tool in the management of PUL, it is rarely reported in Europe and the United Kingdom [22, 34, 37].

Condous et al. [38] prospectively evaluated the risk of inadvertent termination of pregnancy if a uterine curettage would have been performed according to four previously published protocols to define PUL nonviability. The authors found that established criteria for the use of uterine curettage can theoretically result in inadvertent termination of a viable IUP in up to 12.3% of cases. Therefore, they recommended that uterine curettage should not be used in the routine diagnostic workup of women with PULs and should be reserved to patients with persistent PULs. Although rare (<1%) some serious complications such as uterine perforation, hemorrhage and infection have been reported after uterine curettage [32]. Another potential risk associated with uterine curettage is the development of intrauterine adhesions (IUAs) and Asherman syndrome. IUAs have been associated with menstrual disturbances, infertility, and obstetrics complications. Although the true prevalence of IUAs after uterine curettage is not known, they have been reported in up to 21% of patients [39].

### 2.10. Persistent PUL

Persisting PUL, defined as a gestation that started as a PUL and in which *β*-hCG levels fail to decline and no evidence of pregnancy is identified by TVUS accounts for 2% of PULs [22, 31]. These are likely to be either a small EP that has not been visualized or retained trophoblast in the endometrial cavity. This term is not a final diagnosis, and four outcomes have been described [22], depending on the diagnostic or therapeutic interventions performed to achieve a definitive diagnosis (Figure 1): (i) Non-visualized EP: Persistent (plateau) or rising levels of *β*-hCG after uterine evacuation. (ii) Treated persistent PUL: Medical management of PUL without confirmation of the location of the gestation. (iii) Resolved persistent PUL: Spontaneous resolution of *β*-hCG levels with expectant management or after uterine evacuation without evidence of chorionic villi on pathology. (iv) Histological IUP: Chorionic villi identified in contents of uterine evacuation.

The optimal management strategy for persistent PULs is still not established, and several interventions have been described, including expectancy, uterine evacuation, and empirical medical treatment with methotrexate [25, 40]. Currently, the use of diagnostic laparoscopy in order to clarify PUL outcomes leads to many unnecessary surgical interventions, therefore is considered exceptional and should be restricted for symptomatic or hemodynamically unstable patients [29, 41].

In 2014, a small randomized trial failed to demonstrate differences between single-dose methotrexate and expectant management for women with a persisting PUL or EP [42]. However, a more recent clinical trial including 255 hemodynamically stable women with persisting PULs, randomized either to expectant management, active management with uterine evacuation followed by methotrexate if needed or active management with empirical methotrexate, demonstrated that patients who received active management achieved successful pregnancy resolution, without change in their initial management strategy, more frequently [43]. Furthermore, among active management strategies, regarding successful pregnancy resolution without change in management strategy, empirical methotrexate was noninferior to uterine evacuation followed by methotrexate if needed. However, it should be noted that the study had substantial crossover between groups and should be considered when interpreting their results.

## 3. Discriminatory Zone: An Evolving Concept

The term “discriminatory zone” was coined by Kadar et al. [44] in 1981 and refers to the maternal *β*-hCG serum level above which a gestational sac should be visible consistently on ultrasound in a normal IUP. Thus, if the serum *β*-hCG was over the discriminatory level and no intrauterine gestational sac was seen on ultrasound, it should be safe to treat the patient for suspected EP without fear of damaging a normal IUP. Initially, this discriminatory level was set between 6,000 and 6,500 IU/L (using Second IS) among patients evaluated by transabdominal ultrasound. As the resolution of sonography has significantly improved, allowing proper visualization of an intrauterine gestational sac earlier in gestation, the discriminatory *β*-hCG level has been progressively lower and subsequent studies using TVUS have evaluated different cut-off levels ranging between 1,000 and 2,000 IU/L [45–47]. However, the utility of *β*-hCG discriminatory level has been challenged in light of some studies that reports ultrasonography confirmation of IUPs on follow up, when no sac was noted on initial TVUS and the serum *β*-hCG was above the discriminatory zone [48–50].

Connolly et al. [50] evaluated the threshold of *β*-hCG levels associated with the probability of visualization of gestational sacs, yolk sacs, and fetal poles in 651 patients with symptomatic early pregnancies. Despite the threshold values of *β*-hCG at which these structures could be seen were very low, the discriminatory levels at which intrauterine structures would be predicted to be seen 99% of the time were 3,510 IU/L, 17,716 IU/L, and 47,685 IU/L for gestational sac, yolk sac, and fetal pole, respectively. Although improvements in ultrasonography have resulted in lower threshold values for the detection of intrauterine gestational structures, the discriminatory *β*-hCG levels for visualization of such structures are higher than previously reported. If the concept of discriminatory zone is to be used as a diagnostic aid in patients at risk of EP, the value should be conservative high (i.e. 3,500 IU/L) to avoid the potential misdiagnosis and a possible interruption of an IUP. It is important to acknowledge that women with multiple gestations have higher *β*-hCG levels than those with singleton pregnancies at any gestational age and may have *β*-hCG levels above the discriminatory zone before ultrasonography recognition.

### 3.1. *β*-hCG Profile in Ectopic Pregnancy

Silva et al. [51] evaluate *β*-hCG patterns in 200 patients with symptomatic early pregnancies that ultimately were diagnosed as EPs. The median rise in serum *β*-hCG levels was 25% in 2 days. Sixty percent of patients had a rise in *β*-hCG, whereas 40% presented a decrease in *β*-hCG concentrations in 2 days. Among women with rising levels, the median increase was 75% in 2 days (slower than the average for women with viable IUPs). Among women with declining concentrations, the median decrease was 27% in 2 days (slower than the mean decline described for women with spontaneous miscarriage). Nonetheless, 20.8% of women presented with a rise in *β*-hCG values similar to the minimal rise for women with a viable gestation, and 8% of women presented with a fall in *β*-hCG values similar to women with a completed spontaneous miscarriage. The authors concluded that there is no single way to characterize the pattern of *β*-hCG for ectopic pregnancy, and that *β*-hCG profile in women with EP can mimic an IUP or a completed spontaneous miscarriage in approximately 29% of cases. Therefore, although 70% of EPs exhibited patterns of rise or decline outside the “normal” range, the diagnosis of an EP cannot reliably be done based solely on *β*-hCG profile. Other studies have confirmed the overlap of *β*-hCG curves among IUPs, EPs, and spontaneous miscarriages [52].

Several studies have demonstrated that EPs can be managed expectantly in selected populations of stable patients with low *β*-hCG concentrations (<2,500 IU/L) or declining levels [52, 53]. Helmy et al. [54] analyzed *β*-hCG clearance in 266 asymptomatic patients with small nonviable, unruptured EPs, with initial hCG level <5,000 IU/L, who were managed expectantly without need for medical or surgical intervention. Intervention was performed if women presented increasing abdominal pain, or sustained rise of *β*-hCG levels on repeated measurements. All patients were followed on an outpatient basis, until serum *β*-hCG levels were <20 IU/L or urine pregnancy test became negative. Expectant management was successful in 166 (61%) of patients. The median serum *β*-hCG clearance time in this group was 19 days (range 5–82 days). Interestingly, the authors identified two different patterns of *β*-hCG clearance. Seventy-five percent of patients showed a sustained decline at a steady rate from the initial *β*-hCG measurement onwards. On the other hand, 25% of patients showed plateauing *β*-hCG levels for a median of 9 days (range 2–26 days) before starting to decline, which translated into a longer clearance time. These data suggest that the rate of successful expectant management could increase if intervention in patients with nonviable EP is based on clinical symptoms rather than the monitoring of *β*-hCG changes, and that the presence of initial plateauing levels should not be initially considered as an expectant management failure. Nonetheless, it is important to emphasize that women with EPs and decreasing *β*-hCG values should be closely monitored until nonpregnant levels are reached because rupture of an EP can occur while levels are decreasing or are very low [55].

A heterotopic pregnancy refers to the situation when an EP is found simultaneously with an IUP. The incidence in natural conceptions was originally estimated to be 1 in 30,000 pregnancies. However, it seems to be higher (1–3%) in pregnancies achieved through assisted reproductive technologies, and the risk increases in proportion to the number of embryos transferred [29]. To date, no data have been published regarding *β*-hCG dynamics in heterotopic pregnancies, and they constitute an exception to the presented parameters. In heterotopic pregnancies surgery is usually required, and methotrexate is contraindicated. In clinical practice, it is important to remember that visualizing a IUP does not exclude the presence of a further pregnancy elsewhere in the pelvis, especially if the pregnancy is the result of IVF.

### 3.2. *β*-hCG Resolution after Different Treatments. What to Expect?

#### 3.2.1. Uterine Evacuation

As mentioned previously, the use of uterine evacuation is widely used as a diagnostic tool to differentiate between miscarriage and EP in symptomatic patients with PUL, where the diagnosis of spontaneous miscarriage is based on the presence of chorionic villi on histopathology. It should be noted that endometrial biopsy pipelle sampling is not a substitute for standard curettage because of its low diagnostic performance [56, 57]. However, the use of manual vacuum aspiration cannulas has demonstrated to be safe and effective to avoid methotrexate exposure among patients with PULs [33, 58].

Rivera et al. [58] prospectively evaluated *β*-hCG levels after outpatient manual vacuum aspiration in 23 stable patients with nonviable PUL. A decrease ≥50% in *β*-hCG levels within 1-2 days after uterine aspiration was highly predictive of an abnormal IUP. On the other hand, a lower decrease, plateauing or rising *β*-hCG levels after the procedure, as well as the absence of chorionic villi, suggest that the evacuation was incomplete or the presence of a nonvisualized EP, and further treatment is warranted. Although the change at which *β*-hCG is considered to have plateaued is not precisely defined, it would be reasonable to consider levels to have plateaued if they have decreased by less than 10–15% [59]. Patients who have decreased less than 50% require follow-up, and their management should be individualized, as while failed IUP is more frequent, the risk of EP is still high.

#### 3.2.2. Methotrexate Use in Persisting PULs or Presumed EPs

At many institutions, PULs with abnormal *β*-hCG trends are presumed to be EPs and managed empirically with methotrexate. The indications and protocols for the administration of methotrexate are beyond the scope of this review and have been described elsewhere. Briefly, most of the times, a single-dose protocol of 50 mg/m^2^ intramuscular is recommended [18, 59].

Although methotrexate administration is a noninvasive outpatient procedure, further management requires some degree of expertise and training [60]. Pelvic pain has been reported in almost 60% of cases after methotrexate administration and it is not necessarily a synonym of complication. This so-called separation pain is believed to result from tubal abortion and hematoma formation [18, 61]. Therefore, in presence of stable vital signs and serial normal hematocrits, these episodes are generally self-limited and do not warrant surgical intervention [61]. In addition, increasing *β*-hCG levels could be expected in almost 90% of patients after treatment initiation [18, 59], this could be related to the lysis of trophoblastic cells and should not be considered as an abnormal *β*-hCG trend. Therefore, first *β*-hCG quantification must not be obtained sooner than 4 days of methotrexate administration [59]. In clinical practice, *β*-hCG should be measured on the day of administration, and repeated on day 4 and 7, and a *β*-hCG decrease of at least 15% should be expected during this time. Following measurements must be performed weekly until nonpregnant levels are reached. If the decrease between day 4 and 7 is <15%, an additional dose of methotrexate should be considered. During this period vaginal bleeding could be expected, prenatal folic acid supplementation should be discontinued as it may decrease the efficacy of methotrexate, and anti-D immunoglobulin should be considered in Rh-negative patients [43, 62]. Notably, a longer time to pregnancy resolution has been shown in patients with methotrexate treatment compared to patients who underwent uterine evacuation [43] or surgical treatment [63]. Therefore, patients receiving methotrexate should be counseled on the continued risk of EP rupture despite adequate decline of *β*-hCG levels.

#### 3.2.3. Surgical Treatment of EP

Surgical options for the management of EP include salpingectomy or salpingostomy, preferably by laparoscopy. Although a sharp decline in *β*-hCG levels can be expected after salpingectomy, in current clinical practice it is not necessary to follow-up *β*-hCG levels after salpingectomy despite trophoblast spillage had been noted during surgery [64]. Likewise, a longer time until *β*-hCG resolution can be expected when salpingostomy is performed [65].

When salpingostomy is performed, it is important to monitor *β*-hCG levels until they become undetectable [59]. If *β*-hCG levels rise or plateau, the diagnosis of persistent EP is made.

Busacca et al. [66] reported a pronounced fall in *β*-hCG levels on postoperative day 3 for all patients who underwent both conservative and radical surgical treatment. Persistence was diagnosed based on rising or plateauing *β*-hCG levels on postoperative day 7 [66]. Persistent EP can develop in 3–20% of cases after salpingostomy [64], and some studies have proposed a single prophylactic dose of methotrexate after the procedure if there is concern for incomplete trophoblastic removal [66, 67].

## 4. Conclusions

Serial *β*-hCG concentration measurements are widely used to differentiate normal from abnormal pregnancies. Compared to the pattern observed in viable intrauterine pregnancies, *β*-hCG levels increase at a slower rate in most, but not all, ectopic and nonviable pregnancies. Moreover, it is important to acknowledge that observation of a “normal” rise in *β*-hCG does not eliminate the possibility of a miscarriage or EP. Such diagnosis should be sought definitively with a proper correlation and interpretation of *β*-hCG levels with findings at transvaginal ultrasonography or uterine evacuation.

Approximately 95% of patients with miscarriage or spontaneously resolving PULs will have a decrease in *β*-hCG concentrations of 21–35% in 2 days when initial *β*-hCG levels lie between 250 and 5,000 IU/L and 12–21% in 2 days when starting *β*-hCG values are between 50 and 250 IU/L. However, it is important to acknowledge the possibility that these curves may include EPs that spontaneously resolved. It is still not known if the described parameters can be applied to miscarriages resulting from assisted reproductive technologies. Because of the potential for multiple embryos to implant and resolve at different rates it is possible that the expected pattern of decline would change, resulting in an even slower rate of decrease. A woman with decreasing *β*-hCG values and at risk of EP should be monitored until nonpregnant levels are reached because rupture of an EP can occur while levels are decreasing or are very low.

Heterotopic pregnancies are a very rare form of multiple gestations that are nonetheless more likely to occur after IVF. These pregnancies are difficult to diagnose, and no data have been published regarding the observed or expected behavior of *β*-hCG in these cases.

The term PUL is not synonymous with EP, and most patients with PUL are ultimately classified as failed PUL, without risk of significant complications. Whilst the majority of these patients will be failed IUPs, a proportion will be failed EPs without need of further treatment. A small proportion of women may be classified as persistent PUL, which tends to behave biochemically as EPs.

Provided data can be used for clinicians when managing patients with symptomatic early pregnancies either with low *β*-hCG levels or when transvaginal ultrasonography is not conclusive. [68].

## Figures and Tables

**Table 1:** Expected minimal increase (%) in *β*-hCG among normal gestations.

Author, year (References)	*n*	Confidence interval (%)	1 day (%)	2 days (%)
Kadar, 1981 [7]	20	85	29%	66
Barnhart, 2004 [8]	287	99	24%	53
Seeber, 2006 [9]	1249	99.9	NA^*∗*^	35

^*∗*^Not available.

**Table 2:** Expected minimal increase (%) in *β*-hCG among normal gestations according to the initial hCG level.

Initial *β*-hCG (IU/L)	1 day later (%)	2 days later (%)
100	37	84
500	29	64
1000	25	55
1500	23	49
2000	22	46
2500	20	43
3000	19	40
3500	18	38
4000	18	36
4500	17	35
5000	16	33

*Note.* Values represent the first centile and may be used to reflect the minimal “normal” rise. Adapted from Barnhart et al. [8].

**Table 3:** Expected minimum decrease (%) in *β*-hCG during the first week of miscarriage according to the initial *β*-hCG level.

Initial *β*-hCG (IU/L)	Day 2	Day 7
50	12	34
100	16	47
150	18	53
200	19	57
250	21	60
300	22	62
400	23	65
500	24	68
1000	28	74
1500	30	77
2000	31	79
2500	32	80
3000	33	81
4000	34	83
5000	35	84

*Note.* Values represent the 95^th^ centiles and may be used to reflect the slowest “normal” decline. Adapted from Barnhart et al. [17] and Chung et al. [19].

**Table 4:** Expected minimal decrease (%) in *β*-hCG during the first week in spontaneously resolved PULs according to the initial *β*-hCG level.

Initial *β*-hCG (IU/L)	Day 2 (%)	Day 7 (%)
250	35	66
500	38	74
1000	42	79
1500	44	82
2000	46	83
2500	47	84
3000	48	85
4000	49	86
5000	50	87

*Note.* Values represent the 95^th^ centiles and may be used to reflect the slowest “normal” decline. Adapted from Butts et al. [28].

## Data Availability

No underlying data were collected or produced in this study.
